# Aging-Related Molecular Pathways in Chronic Cholestatic Conditions

**DOI:** 10.3389/fmed.2019.00332

**Published:** 2020-01-21

**Authors:** Claudio Pinto, Elisabetta Ninfole, Antonio Benedetti, Luca Maroni, Marco Marzioni

**Affiliations:** Department of Gastroenterology and Hepatology, Università Politecnica delle Marche, Ancona, Italy

**Keywords:** aging, PSC, PBC, senescence, therapeutics, inflammation, fibrosis

## Abstract

Aging is commonly defined as the time-dependent functional decline of organs and tissues. Average life expectancy has increased considerably over the past century and is estimated to increase even further, consequently also the interest in understanding the aging processes. Although aging is not a disease, it is the major risk factor for the development of many chronic diseases. Pathologies, such as Primary Biliary Cholangitis (PBC) and Primary Sclerosing Cholangitis (PSC) are cholestatic liver diseases characterized by chronic inflammation, biliary damage and ultimately liver fibrosis, targeting specifically cholangiocytes. To date, the influence of aging in these biliary diseases is not fully understood. Currently, liver transplantation is the only solution because of lacking in efficiently therapies. Although liver cells have a high regenerative capacity, they undergo extensive molecular changes in response to aging. Following time-dependent damage induced by aging, the cells initially activate protective compensatory processes that, if hyperstimulated, can lead to the decline of regenerative ability and the development of pathologies. Recent studies have introduced novel therapeutic tools for cholangiopathies that have showed to have promising potential as novel therapies for PSC and PBC and for the development of new drugs. The recent advancements in understanding of molecular aging have undoubtedly the potential to unveil new pathways for selective drug treatments, but further studies are needed to deepen their knowledge.

## Introduction

The aging of a biological system is the inevitable process determined by time-dependent accumulation of damage to genetic material (1) that commonly involves DNA damage, telomere shortening, and epigenetic alterations. The functions of organs inevitably decline in time, leading to body deterioration, and increased susceptibility to death (2, 3). Average life expectancy has increased dramatically over the past century and is estimated to increase even further. However, the sharp increase in the number of elderly people suffering from chronic diseases suggests that an increase in life span does not necessarily coincide with a prolonged health span. The aging research goal focuses mainly on improving health span, given the health costs associated with the years of oldness. Although aging is not a disease, it is the major risk factor for the development of many chronic diseases (4–6), in particular chronic liver conditions (7, 8). Specific age-related hepatic changes have already been highlighted and may affect liver morphology, physiology and oxidative capacity, besides affecting the regenerative capability. Old age seems to favor non-alcoholic fatty liver disease (NAFLD), NASH, and ultimately HCC, principally caused by an increased inflammation in agreement with the inflamm-aging theory. Pathologies affecting cholangiocytes (cholangiopathies), such as Primary Biliary Cholangitis (PBC) and Primary Sclerosing Cholangitis (PSC), are cholestatic liver diseases (targeting intra- and extra-hepatic cholangiocytes), characterized by chronic inflammation and thus biliary damage which cause proliferation and ultimately liver fibrosis, which develop and progress differently according to the patient age. Over the years, more data have been obtained regarding PSC. This pathology is characterized by destruction of intrahepatic/extrahepatic bile ducts, chronic biliary inflammation, liver fibrosis (9, 10) and is often associated with inflammatory bowel disease rising risks of developing colorectal cancer and cholangiocarcinoma (11–13). PBC, is an autoimmune disorder that specifically target cholangiocytes, characterized by the injury of small- and medium-sized bile ducts, cholestasis, and lymphocyte infiltration (14–16). Has been shown that PBC predominantly affects women, with higher incidence in patients who have a relative with PBC or any other autoimmune disorder (17). Currently, liver transplantation is the only solution for cholangiopathies because of lacking in efficiently therapies. Although new preclinical studies have provided attractive prospects for the development of new therapeutic approaches, especially for PSC, further investigation is needed in understanding mechanisms and pathophysiology of PBC to identify new candidate targets.

## Hallmarks of Aging

Studies carried out over the years to understand typical aging processes have led to the identification of nine hallmarks that can be grouped into three main categories (3). The primary hallmarks are the cause of age-related damage, such as genomic instability, telomere attrition, epigenetic alterations and loss of proteostasis (18, 19). The response to these age-related damages, named antagonistic hallmarks, include cellular senescence, deregulated nutrient sensing and altered mitochondrial function. Finally, integrative hallmarks are the consequence of responses and responsible of aging phenotype which leads to stem cell exhaustion and altered intercellular communication.

## Primary Hallmarks

The accumulation of both genomic and mitochondrial DNA damage depends on exogenous stressors (physical, chemical or biological triggers) and/or endogenous events (DNA replication errors or ROS production) (1, 20). The oldest cells are the ones with the higher genomic instability. Typical of old cells are telomers mutations and their length has been shown to be highly heritable (21). The correlations between telomeres shortening and aging has been demonstrated in different animal models (22) and in several age-related diseases (23–25). DNA methylation (epigenetic alteration that principally involve CpG islands) seems to be a predictor of human age in genome-wide methylation studies (26–28). The impairment of proteostasis (principally due to toxins or free radicals), lead to the chronic expression of unfolded or misfolded protein or to the accumulation of protein aggregates, process that has been linked to different age-related pathologies of nervous system (29–31).

## Antagonistic Hallmarks

Cell activates compensatory processes known as antagonistic hallmarks, in response to the primary hallmarks. They are initially protective processes but, when hyperstimulated, may lead to cellular aging or development of pathologies. Cellular senescence (defined as the irreversible arrest of cell growth) represents the main response to age-related damage. Senescence is associated with complex cellular changes, such as chromatin reorganization, metabolic reprogramming, increasing of autophagy and release of proinflammatory mediators and growth factors known as senescence-associated secretory phenotype (SASP) (32). Cellular senescence is not exclusively associated with aging but occurs in response to multiple inducing factors, remodeling the tissue in order to solve the damage. *In vivo* data have shown the accumulation of senescent cells in aged tissues (33, 34). The lack of balance between clearance of senescent cell and mobilization of progenitor cells, determines the accumulation of senescent cells, which contributes to aging. Nutrients sensing deregulation and mitochondrial dysfunction are also common with advancing age. The main physiologic pathway affected by aging process, in both humans and model organisms, is the growth hormone (GH)/insulin like growth factor (IGF-1) axis, that lead to impaired glucose sensing (35, 36). Others nutrient sensing systems involved in the detection of cellular energy status, such as AMPK (which detect high AMP levels) and Sirtuins (which detect high NAD^+^ levels) may also play important role in aging processes (37). Finally, aging-related mitochondrial dysfunction has been associated with deletion of mtDNA, oxidation of mitochondrial proteins, destabilization of the macromolecular organization of respiratory chain complexes, alteration of lipid composition of mitochondrial membranes, defective mitophagy and imbalance between fission and fusion events (38, 39).

## Integrative Hallmarks

As the organism ages it decreases the regenerative ability of the tissues because of depletion of stem cells niches and changes in intercellular communication (i.e., endocrine, neuroendocrine, or neuronal). For example, it is known that mesenchymal stem cell decline leads to osteoporosis, haematopoietic stem cell exhaustion results in a less production of adaptive cells (called immunosenescence) that leads to anemia and intestinal epithelial stem cell depletion causes decreased intestinal function (3). Immunosenescence and increased secretion of cytokines by adipose tissue lead to chronic inflammation (40, 41). Chronic low-grade systemic inflammation combined with immunosenescence are part of the pathogenesis of premature aging, also called inflammaging. Another physiological change that negatively influences liver function is the redistribution of adipose tissue from subcutaneous to visceral sites (42). This observation, together with the decline of immune system efficiency, accumulation of senescent cells, inflammaging, and defective autophagy, increase mortality and risk of disorders, such as hypertension, atherosclerosis, hyperlipidemia, insulin resistance, and diabetes, all of which predispose to developing NAFLD (40, 43).

## Molecular Mechanisms of Aging in Liver Disease

The liver is a pivotal organ with a wide range of functions, including detoxification, protein synthesis, regulation of energy metabolism and much more. Specific age-related hepatic changes have been reported, such as enhanced hepatocyte size, increase in the number of binucleated cells, reduction in mitochondrial number, excessive visceral fat and secretion of pro-inflammatory cytokines (44–46). These changes significantly affect liver morphology, physiology, and oxidative capacity. At molecular level, aged livers go through the loss of the regenerative capacity and may involve CCAAT/enhancer-binding protein (C/EBP) family members, glycogen synthase kinase 3 Beta (GSK3β), histone deacetylase 1 (HDAC1), and Sirtuin 1 epigenetic and signaling pathways (47–51). Age-related accumulation of lipids in the liver has also been reported (52). The resulting lipotoxicity increase the prevalence of NAFLD in elderly person (53). Furthermore, aging significantly enhance the progression to NASH and fibrosis, thus predisposing to increased mortality in elderly subjects with NAFLD (54, 55).

## Liver Endothelial Sinusoidal Cells

**Figure 1:**
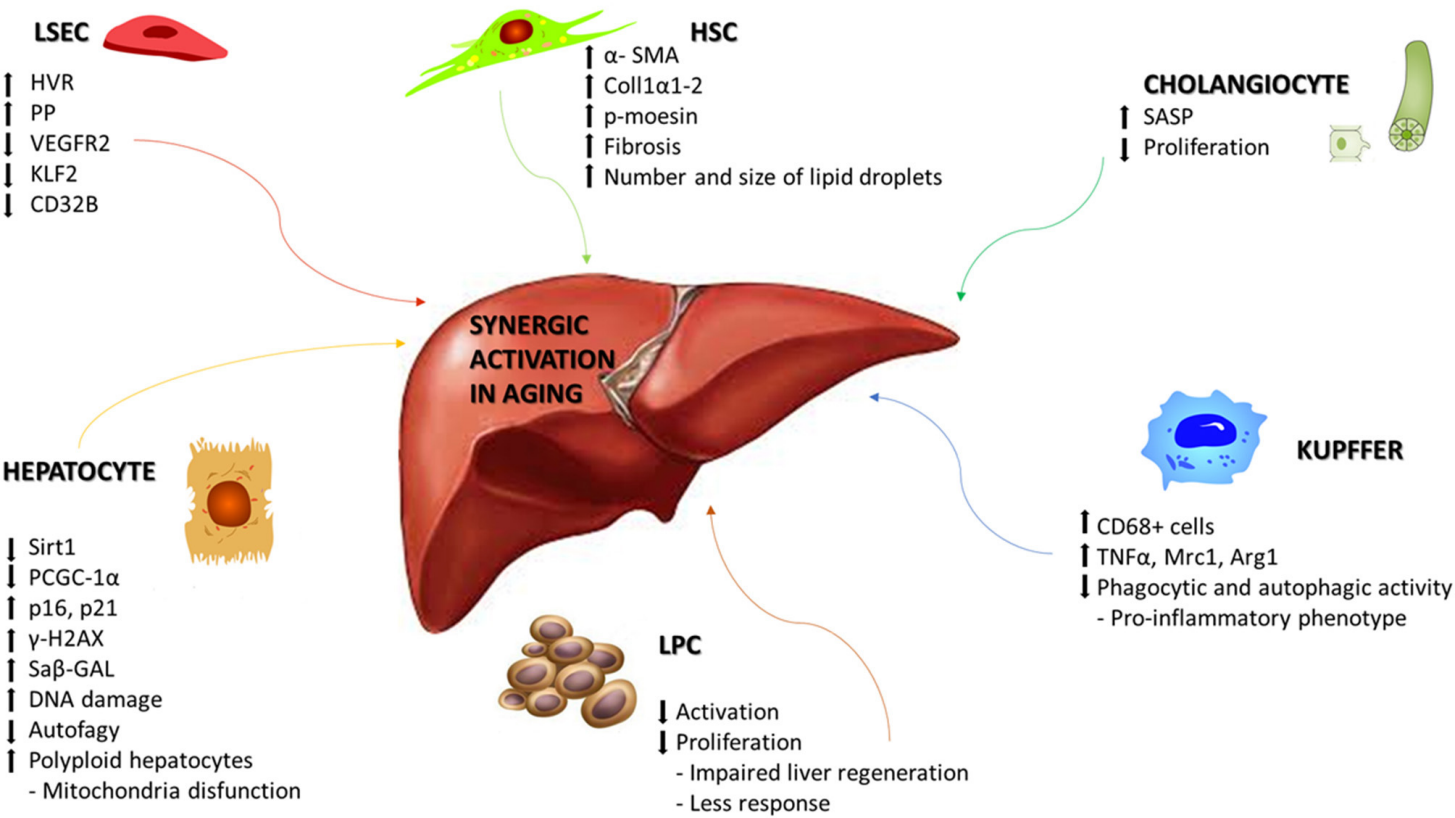
Synergic activation of liver cells in aging. Schematic representation of the main molecular mechanisms associated with aging in liver disease. Age-related changes can include alterations that affects morphology, physiology, and oxidative capacity in the different cellular populations of the liver. HVR, Hepatic vascular resistence; PP, Portal pressure; VEGR2, Vascular endothelial growth factor receptor 2; KLF2, Krüppel-like Factor 2; Sirt1, Sirtuin1; PGC-1α, Peroxisome proliferator-activated receptor gamma coactivator 1-alpha; γ-H2AX, H2A histone family member X; Saβ-GAL, Senescence-associated beta-galactosidase; TNFα, Tumor necrosis factor alpha; Mrc1, Mannose receptor, C type 1; Arg1, Arginase 1; SASP, Senescence-associated secretory phenotype; α-SMA, Alpha smooth muscle actin; Coll1α1-2, Collagen 1α1-2; p-moesin, Phosphorylated moesin.

At the level of single-cell populations a recent study demonstrated that aging is associated with sinusoidal remodeling, both in rodents and humans (56, 57). LSECs are endothelial cells that line the hepatic sinusoids, whose main role is to facilitate bidirectional exchanges between blood and hepatocytes. LSECs also mediate endocytosis of circulating proteins, having a role in the regulation of immunotolerance, and maintaining sinusoidal microenvironment. Old rats exhibited significantly higher hepatic vascular resistance *in vivo*, with reduced liver perfusion and increased portal pressure in comparison with young ones. From a molecular point of view, sinusoidal pseudocapillarization is associated with reduced expression of VEGFR2, KLF2, and CD32b, altered expression of the von Willebrands factor, CD31 and collagen (58), and so, in a reduction in the number and size of fenestrations, thickening of the endothelium, deposition of basal lamina and collagen (56, 57) (Figure 1). Due to these alterations, the lipoproteins and insulin absorption is compromised causing hyperlipidemia and hepatic insulin resistance (59).

## Hepatocytes

Age-related molecular alterations induce a reduction in the number of hepatocytes but with an increased portion of polyploid hepatocytes, along with reduced rates of DNA synthesis and repair (60). Hepatocytes, the major parenchymal cells in the liver, are the chief functional cells of the liver and perform metabolic, endocrine and secretory functions as well as protein synthesis, detoxification, activation of innate immunity and so on. Dysregulation of glycolysis, triglyceride synthesis, and lipid metabolism occurs because of decreased expression of Sirtuin1, PGC-1α, lower concentrations of NAD^+^, and upregulation of the senescence marker p16 (61). On the other side, hepatocytes show to be relatively resistant to telomer shortening (62), maybe due to the high expression levels of telomerase (63). Other molecular modifications have been associated at hepatocytes aging, such as increased heterochromatin protein 1β, elevated senescence-associated-β-galactosidase activity, p21, p16, and γ-H2AX (64), but also genes involved principally in hepatic metabolism of glucose, lipids and proteins, such as PI3K/Akt, MAPK, Jak/S, NF-κB, TGFβ, IGF1, and Ca^2+^/cAMP (65). Alterations in mitochondrial biogenesis and autophagic degradation (mitophagy) have been observed together with the presence of enlarged mitochondria (66, 67) and the reduction in hepatocytes autophagy in old livers, underlined by lowering number of autophagic vesicles (68, 69) (Figure 1).

## Hepatic Stellate Cells and Kupffer Cells

Aging affects also hepatic stellate cells (HSC) and Kupffer cells (KC). HSCs are pericytes located within the space of Disse and maintain close interactions with sinusoidal endothelial cells and hepatic epithelial cells. HSCs are involved in vitamin A and lipid storage, when activated, they acquire their characteristic phenotype and produce collagen, starting the development of hepatic fibrosis. An increased expression of HSC activation markers, such as αSMA, collagen 1α1-2, and phosphorylated moesin, has been described in aged rats (70, 71). The lipid droplets in HSC significantly increase in number and size during aging, as observed in mice and non-human primates thus starting the development of hepatic fibrosis (72, 73). KCs are specialized macrophages located in the liver, lining the walls of the sinusoids, with the main function of performing phagocytic functions to remove cellular debris from the portal blood flow. KC activation is observed in most types of liver diseases and contributes to the pro-inflammatory status of the hepatic sinusoid. Phagocytic and autophagic activity of KC decline with aging, acquiring an inflammatory phenotype (71). In aged rats is increased the infiltration of CD68^+^ cells along with significant differences in the mRNA expression of cytokines including TNFα, Mrc1, and Arg1 (70) (Figure 1).

## Liver Progenitor Cells

Liver progenitor cells (LPCs) are quiescent cells that are activated during liver injury in order to regenerate the liver parenchyma. LPC functionality is negatively regulated by the aging process (74). LPCs in young mice could be activated and proliferate upon liver injury, whereas in old mice failed to respond and proliferate, leading to impaired liver regeneration. Levels of Reactive Oxygen Species (ROS) and neutrophils infiltration are increased in aged mice, in collaboration with chemokine production from activated HSC and decrease activation and proliferation of LPC (Figure 1).

## Cholangiocytes and Cholangiopathies: Focus on PSC and PBC

Cholangiocytes are the cells lining the biliary tract and the target of cholangiopathies, such as PSC and PBC. PSC is an idiopathic, autoinflammatory disorder characterized by fibrosis and obliteration of medium and large ducts throughout the biliary epithelium. In 70% of patients is associated with inflammatory bowel disease, particularly ulcerative colitis, and it may progress in complications, such as cholestasis, hepatic failure, autoimmune disease, and cholangiocarcinoma (75, 76). PBC is an autoimmune liver disease that predominantly affects women [maybe due to the observation that 48% of PBC patients experienced prior recurrent urinary tract infections, more frequent in women and caused by *Escherichia coli* (77)], characterized by mainly portal inflammation, chronic cholestasis and destruction of small intrahepatic bile ducts that show a progressive pathogenesis from liver fibrosis to cirrhosis, portal hypertension and ultimately liver failure (78, 79). Cholestatic liver diseases are profoundly influenced by patient age. A more severe disease course in young patients affected PBC has been recently observed in a large retrospective study, showing increased risk of treatment failure, liver transplantation and death (80). In PSC, the age at diagnosis increase the risk of develop cholangiocarcinoma (21% for patients older than 60 years) (81). In elderly patients is risen the risk of complications after liver transplantation. The development of biliary complications after orthotopic liver transplantation is influenced by donor age (82), and lower survival rates in people aged more than 60 years (5-years survival rate of 59%) has been observed (83). However, the survival of recipients older than 70 years of age still remains lower than in younger patients (10-years survival of 43 vs. 64% in recipients aged 60–69 years) (84).

Important clues supporting the role of aging in shaping cholangiocyte biology in course of biliary injury are also emerging. The analysis of liver samples collected from PSC and PBC patients have shown increased expression of senescent markers and SASP components in diseased cholangiocytes (85). Furthermore, has been found a significantly increased expression of N-Ras protein co-localization with activated RAS in PSC, which was absent in PBC or control samples. These data underline the role of N-Ras protein as mediator of lipopolysaccharide-induced inflammation, further supporting a potential role for N-Ras signaling in the pathogenesis of PSC (86). In PBC seems to be more important the role of the autophagy as a necessary component to the activation of cell senescence (87). Senescent PBC cholangiocytes accumulated markers of autophagy, such as microtubule-associated proteins-light chain 3β, cathepsin D, and lysosome-associated membrane protein-1 (88). Several studies have shown as deregulated autophagy might be involved in the induction of cholangiocyte senescence in several biliary disease. The expression of autophagy markers as LC3 and p62 is significantly correlated with the expression of the known senescent markers, p16 and p21, in ductular cells in ductular reaction (DR) (89). In early and advanced stages of PBC, autophagy is frequently correlated with cellular senescence in bile ductular cells in DRs. Sasaki et al. suggests that autophagy may be involved in the pathophysiology of DRs in PBC and may precede the cellular senescence. Moreover, they have also found that deregulated autophagy may contribute to the abnormal expression of mitochondrial antigens and be involved in the autoimmune pathogenesis of bile duct lesions in PBC (89–91).

These findings are corroborated by data obtained in animal models of cholestatic liver injury. Isolated cholangiocytes from multi-drug resistance 2 knockout (Mdr2^−/−^) mice develop cellular senescence (92). As a direct consequence of Mdr2 lack of expression, toxic bile acids accumulate in the bile triggering increased bile duct mass and liver fibrosis. Despite these findings demonstrate a link between senescence and disease presentation, it is unknown whether senescence is the trigger of disease or if it is a consequence of chronic damage (93, 94).

## New Potential Pathways Involved in PSC and PBC For Novel Therapies

In order to identify new molecular pathways involved in course of cholangiopathies are needed adequate preclinical animal models to mimic, as much as possible, the features of relative human diseases (95–97). To date, for PSC and PBC, various genetic and chemical models are used in parallel with *in vitro* studies to resemble the pathogenesis of these pathologies (98). PSC is a heterogeneous disease, the identification of clinical endpoints and treatment goals in PSC remains difficult to determine for the complex interaction of multiple causes, such as environmental insult (99, 100), genetic susceptibility (101), dysregulation of immune signaling (102, 103) and gut microbiome derangement (104–106) (Table 1). New drugs that act selectively at the level of senescent cells are being evaluated for a series of human diseases, such as senolytics, inhibitor of the anti-apoptotic proteins BCL-2 and BCL-xL, inhibitor of complex that modulates SASP production (mTORC1 and JAK2/STAT3 pathway) and other alternative approaches (125). As suggested by Zhou et al. a possible pharmacological target for the treatment of cholangiopathies can lie among the mediators of Secretin/SecretinReceptor axis (107) and secretion pathway of TGF-β1 at the biliary ducts level (108), both responsible of the biliary damage and liver fibrosis regulation. In this way, another new potential target is the Substance P, a neuropeptide that plays an important role in regulating hepatic fibrosis and cellular senescence (109). In the contest of PSC and PBC, Forkhead Box A2 (FoxA2), a key transcriptional factor involved in tissue regeneration, was found upregulated in LPC and downregulated, through epigenetic mechanisms, in liver tissue (117). This reduction was associated with an exacerbation of fibrotic liver damage suggesting that, acting on an up regulation of FoxA2, could be a therapeutic strategy to reconstruct the hepatobiliary system, after a hepatic injury (Table 1). Recently, has been shown the role of mast cells (MCs) also in course of cholangiopathies. MCs was found surrounded bile ducts during the early stages of PSC but were located in fibrous septa in late-stage PSC (126). Their role in PSC have been studied founding that MC number and markers are increased in Mdr2^−/−^ mice and PSC patients compared with controls (118). Treatment with cromolyn sodium, a MC stabilizer that blocks the release of histamine, reveled a reduction in MC indicators and PSC-associated fibrosis. Furthermore, MCs and their mediators may influence the function of cholangiocytes and hepatic bile production and flow (118). Cholangiocytes secrete also stem cell factor, which is a chemoattractant for c-kit expressed on MCs. Stem cell factor was found increased in human PSC and in Mdr2^−/−^ mice (119). Blocking biliary stem cell factor decreased MC migration, biliary proliferation/senescence, and HSC activation, so targeting MC infiltration may be an option to ameliorate PSC progression (119). In PBC there are minor evidence of MCs role, but is known that PBC patients often presents increased circulating bile acid pools, and it has been demonstrated that specific bile acids can alter MC activation *in vitro* (120, 121). A recent study showed that MCs are located in the portal areas and sinusoidal walls in patients with PBC and an increased expression of chymase that seems to be co-localized in areas that exhibited extensive hepatic fibrosis (127). Despite all these studies demonstrate the increased presence of MCs, and their potential in developing pharmacologic therapies, the causal effect of MCs remains to be fully examined. Other approaches concern the use of Bile acids that are already largely used in cholangiopathies therapy. Ursodeoxycholic acid (UDCA) inhibits cholangiocyte proliferation and secretion *in vivo* (114) and is an approved drug for PBC (115). UDCA stimulates secretion of bile acids from hepatocytes, preventing hepatocyte injury, apoptosis and necrosis and subsequent inflammation and fibrosis. UDCA expand the bile acid pool and induces a less toxic bile composition through the activation of AE2 transporters (116). Furthermore, UDCA administration inhibits MC activation improving liver conditions in Mdr2^−/−^ mice (128), but its use for PSC treatment provided controversial results (129) (Table 1). Instead, there are better perspectives regarding the Obeticholic acid, which is a synthetically modified bile acid known to be a potent Farnesoid X receptor (FXR) agonist. A trial of obeticholic acid for PBC patients has demonstrated to improve serum levels of ALP and bilirubin compared to the placebo group, and long-term clinical outcomes in PBC patients (110, 111). In a clinical trial (NCT02177136), administration of obeticholic acid improved serum ALP and bilirubin levels also in PSC patients compared to the placebo group.

**Table 1:** This table summarize both pathways and group of therapies that are currently under investigation.

**Disease**		**References**
PSC	**Features**	
	- Environmental insults	(99, 100)
	- Genetic susceptibility	(101)
	- Dysregulation of immune signaling	(102, 103)
	- Gut microbiome derangement	(104–106)
	**Potential pathways**	
	- Mediators of Secretin/Secretin receptor axis	(107)
	- Secretion pathway of TGF-β1 at biliary ducts level	(108)
	- Regulation of substance P	(109)
	**Therapies**	
	- Obeticholic acid	(110, 111)
	- Norursodeoxycholic acid, oral antibiotics, such as vancomycin and rifamixim, FXR agonist, LUM001, anti-fibrotics agents, Simtuzumab, and Cenicriviroc	(112)
PBC	**Potential pathways**	
	- Immunosuppressive and immunomodulatory agents	(113)
	**Therapies**	
	- Ursodeoxycholic acid	(114–116)
	- Obeticholic acid	(110, 111)
PSC and PBC	**Potential pathways**	
	- Forkhead Box A2 (FoxA2)	(117)
	- Mast Cells (MCs)	(118–121)
	- Melatonin	(122)
	- Neurokinin 1 receptor	(123)
	- Twinfilin 1	(124)

Others new potential pathways involved in PSC and PBC concern the Melatonin, neurokinin-1 receptor, and twinfilin-1. It has been shown how these molecules may play a role in cholangiocytes response to injury and liver fibrosis (122–124), but further studies are needed and must be deeply investigated (Table 1).

To date, for PSC treatment, various therapies are under investigation. Just some of this [better elucidate in a review of Rodriguez et al. (112)] include, Norursodeoxycholic acid, oral antibiotics, such as vancomycin and rifamixim, FXR agonist, LUM001, anti-fibrotic agents, Simtuzumab and Cenicriviroc. For PBC, less is known, but actually, there is good evidence concern the use of the glucocorticoid Budenoside, fibrates that act as ligand for the nuclear receptor PPAR, fenofibrates and bezafibrate, and other strategies aiming at the availment of immunosopressive and immunomodulatory agents (113) (Table 1). Noteworthy is a new class of drugs that aim to destroy senescent cells, called senolytics (130). The notions about these molecules are still few and need to be further tested on primates and humans, but could have great potential in improving healthspan, given the known accumulation of senescent cells in aging.

## Conclusions

Cholangiopathies are deeply influenced by the aging process, and elderly patients require a careful management in clinical practice. Average life expectancy is constantly increasing, it becomes essential to understand the molecular basis of age-related modifications that are involved in disease progression. Current therapeutic approaches utilize agonists or antagonists to regulate signaling pathways involved in cholangiocyte response, particularly focused on the improve of portal fibrosis and liver inflammation and on the activation of other liver cells by cholangiocytes, such as HSCs and KC leading to further liver damage. Recently, the use of stem cells or stem cell-derived extracellular vesicles has also taken hold and together with the recent studies described above, have showed to have promising potential as novel therapies for PSC and PBC and for the development of new drugs. Such studies have undoubtedly the potential to foster a better management of patients, but further studies are needed to deepen their knowledge.

## Author Contributions

This work was done in full by CP. EN has processed the image and part of the contents. AB and LM have contributed to a part of the contents. MM has carried out the revision and correction of the draft.

### Conflict of Interest

The authors declare that the research was conducted in the absence of any commercial or financial relationships that could be construed as a potential conflict of interest.
